# Knowledge and practice of evidence-based mild traumatic brain injury guidelines among physicians providing emergency and trauma care in Israel

**DOI:** 10.1186/s13584-026-00784-x

**Published:** 2026-09-15

**Authors:** Barak Levit, Tamar Cafri Oppenheim, Forat Swaid, Wissam Najar, Gilad Sorek

**Affiliations:** 1 Tzafon Medical Center, Trauma Unit, Poria, Israel; 2 Department of General Surgery, Tzafon Medical Center, Poria, Israel; 3 Department of Physical Therapy, Faculty of Health Sciences, Ariel University, Ariel, Israel; 4 Tzafon Medical Center, Helmsley Rehabilitation Center, Physiotherapy Department, Poria, Israel

**Keywords:** Mild traumatic brain injury, Concussion, Trauma, guidelines, Emergency department, Israel

## Abstract

**Background:**

Over the past decade, clinical guidelines for the management of mild traumatic brain injury (mTBI) have evolved worldwide to prevent re-injury and reduce the risk of prolonged recovery. However, to date, no study has examined the knowledge and practice of these clinical guidelines in Israel.

**Aims:**

To describe knowledge and implementation of up-to-date post-mTBI guidelines among physicians providing emergency and trauma care in Israel.

**Methods:**

An exploratory cross-sectional survey of 68 hospital-based physicians providing emergency and trauma care (32% residents; 79% male; 87% general surgery/trauma/emergency medicine). Participants completed a 30-item multiple-choice questionnaire covering (1) demographics/professional characteristics and (2) knowledge and implementation of post-mTBI recommendations (e.g., symptom recognition, accepted protocols, and follow-up advice).

**Results:**

Most respondents (90%) recognized common post-mTBI symptoms, such as headaches and dizziness; however, only 40–60% identified neck pain, sleep disturbances, balance problems, and light/noise sensitivity. Although 50% reported familiarity with the internationally accepted guidelines, only 38% instructed for return-to-activity/sport protocol, 32% for return-to-school/work protocols, and 21% provided standardized discharge handouts. While 60% recommended post-injury medical follow-up, only 44% instructed patients to seek further care if they develop persisting symptoms, only 16% referred patients to clinicians specializing in post-mTBI management when clinically indicated, and only two respondents (3%) referred patients to a specialized mTBI clinic. The most frequently reported barrier (38–50%) was a lack of awareness of mTBI specialized services/providers.

**Conclusions:**

In this exploratory sample, gaps in both knowledge and implementation of internationally accepted post-mTBI guidelines were observed among Israeli physicians providing emergency and trauma care. The findings support revisions to national procedures, targeted training, and the development of a coordinated stepped-care pathway that links emergency and primary care with community-based follow-up for all patients following mTBI. They also support better access to interdisciplinary rehabilitation services for those with persistent or complex symptoms.

**Supplementary Information:**

The online version contains supplementary material available at https://doi.org/10.1186/s13584-026-00784-x.

## Introduction

Traumatic brain injury (TBI) is the leading cause of death and of acquired sensorimotor, cognitive, and/or behavioral disability in children and young adults [1]. The overall global annual incidence of TBI is 349 cases per 100,000 people. Approximately 70–80% of adult cases and 80–90% of pediatric cases are classified as mild TBI (mTBI) [2–4]. According to the recent American Congress of Rehabilitation Medicine diagnostic criteria [5], the diagnosis of mTBI requires a biomechanically plausible mechanism of injury and one or more of the following: at least one clinical sign; at least two new or worsened acute symptoms together with at least one clinical or laboratory finding; or neuroimaging evidence of TBI. The “mild” diagnosis applies when loss of consciousness does not exceed 30 min, Glasgow Coma Scale is 13–15 after the injury, and post-traumatic amnesia does not exceed 24 h. The term “concussion” may be used interchangeably with mTBI when neuroimaging is normal or not indicated [5]. Clinically, mTBI is characterized by a wide range of signs and symptoms, such as physical problems, cognitive impairments, psychological–emotional complaints, and sleep disturbances [2, 5–7]. In most cases, symptoms resolve spontaneously within hours to days. However, in 20–30% of cases, complaints persist beyond the initial phase (beyond 4 weeks) and significantly limit the return to regular activity. In those cases that suffer from persistent post-concussion symptoms (PPCS), comprehensive, interdisciplinary care is required [2, 5–7].

Over the past decades, there has been a significant increase in research and treatment following mTBI, leading to changes in accepted guidelines for prevention, diagnosis, and management [5–8]. Current recommendations in the acute stage following mTBI include relative rest for the first 24–48 h, and if no concerning problems arise, a gradual, symptom-limited return to activity can begin after 24–48 h according to return-to-sport/activity protocols, with accommodations for returning to work or studies as needed [5–9]. If symptoms and complaints do not resolve spontaneously (beyond 4 weeks), patients should be referred to interdisciplinary care in a dedicated mTBI clinic [2, 5–9]. These recommendations for a graded return to activity are crucial in preventing repeat head injuries and reducing the risk of prolonged recovery and chronic symptoms [7, 8].

Studies from North America and Europe have shown that emergency department (ED) medical teams lack awareness of the need for immediate management and referral for follow-up care [10–15]. Guidelines are not always operational in centers, and reported policies systematically diverge from the recommendations outlined in the updated guidelines [10]. In addition, patients following mTBI were unlikely to receive proper discharge education [14, 16].

National epidemiological data from Israel suggest that the proportion of mTBI among all TBI cases is lower than reported internationally, with approximately 70% in children and 55–77% in adults [17, 18]. A likely explanation is under-recognition of mTBI in the ED. In addition, a common mechanism of mTBI in North America is organized, high-contact sports [19–21]. The high visibility of sports-related concussion has driven public, media, and professional awareness. It has promoted the development and implementation of structured return-to-sport and return-to-learn protocols [6]. In contrast, in Israel, mTBI is more frequently related to road traffic collisions, falls, and other non-sport mechanisms [18], and sports-related concussion currently has a much lower profile in public discourse and health policy. Moreover, Israeli Ministry of Health circulars on acute management of mTBI do not fully align with current international guidelines, including circulars 50/1999 (“Clinical guidelines for mTBI and concussion”) and 40/2014 (“ED management of mild head injury in infants and children”). Both primarily address risk stratification in the ED, selective use of neuroimaging, short-term observation, and discharge warnings. However, they do not include the most up-to-date return-to-activity protocols and do not emphasize necessary post-discharge treatment when needed. Based on an extensive literature review, no studies were found that examined the knowledge and practice of current clinical guidelines for the acute management and referral for follow-up care of individuals after mTBI in Israel. Such data is a necessary first step toward assessing the need for policy change and developing targeted education and training programs in this field.

This study aimed to describe knowledge and implementation of up-to-date clinical guidelines for discharge instructions and referral to follow-up care after mTBI, among physicians providing emergency and trauma care in Israel.

## Methods

### Participants

An exploratory cross-sectional survey of Israeli physicians providing emergency and trauma care was conducted. The study protocol was approved by the ethics committee at Tzafon Medical Center (0103-22-POR). Because the anonymous survey was distributed through professional networks, physicians participated in their personal capacity, not as representatives of their hospitals, which were not designated as study sites. Before answering any questions, all participants read an information statement and provided informed consent to participate in the study.

The inclusion criteria included physicians working in Israeli hospitals who provide acute care for patients with mTBI (typically within 24–48 h post-injury) in emergency departments and/or trauma-related acute-care settings, and who manage at least one mTBI case per week, including both pediatric (ages 0–18) and adult (ages 18+) patients.

### Survey development

The study team, including the trauma unit at Tzafon Medical Center and two physical therapists, all with expertise in mTBI diagnosis and management, established the questionnaire’s length, format, and content. The questionnaire was developed in accordance with current up-to-date clinical guidelines [5, 7, 9], and with previous studies that investigated similar content in various locations worldwide [12–14, 22, 23]. The questionnaire includes 30 multiple-choice questions and consists of two parts: (1) general details (for example, age, gender, place of work), and (2) knowledge and implementation of up-to-date clinical guidelines for acute management and referral to follow-up care following mTBI. Four domains were identified in this section: (1) knowledge of different symptoms and risk factors after mTBI; (2) knowledge and utilization of existing mTBI guidelines and recommendations; (3) initial management using gradual return-to-sport/school/work protocols; and (4) referral to follow-up care. The time required to complete the survey was approximately 10–15 min. The complete questionnaire is available in *Supplemental Material*.

### Data collection

Data collection took place from December 2024 to November 2025 using an online questionnaire platform in Google Forms, distributed via professional social media networks in trauma and emergency medicine and in Israel. The main distribution channel was a national trauma WhatsApp group with 203 members. The survey was initially shared with adult and pediatric trauma and emergency department physicians nationwide and subsequently circulated to their teams within their hospitals. During the study period, 4–5 reminders were sent through social media networks. Responses were recorded using a dedicated Google Forms instrument created specifically for this study. The data collection file was developed and approved for security by the Tzafon Medical Center Information Security team. All responses were anonymous, and no personally identifying information was collected. Because dissemination relied on professional networks and onward sharing, findings should be interpreted as exploratory rather than nationally representative.

### Statistical analysis

Descriptive statistics were used to summarize the characteristics of respondents and questionnaire responses. Differences in survey outcomes by patient population (physicians treating children vs. adults), professional seniority (residents vs. senior physicians), and geographic practice setting (central Israel vs. peripheral regions) were explored using a chi-square test or a Mann–Whitney test, as appropriate. Given that three factors were tested (patient population, professional seniority and geographic practice setting), statistical significance was set at *p* < 0.012 (i.e., 0.05/3). Statistical analyses were performed using SPSS version 29 (SPSS Inc., Chicago, IL, USA).

## Results

Of the 70 physicians who responded to the survey, 68 reported seeing at least one individual per week following mTBI and were included. Respondents’ characteristics, including demographic information, medical expertise, and years of experience in diagnosing and treating individuals following mTBI, are presented in Table 1.

**Table 1:** Demographic characteristics of survey respondents

Age (years)	25-30		5 (7)
	31-40		25 (37)
	41-50		27 (40)
	51-60		9 (13)
	>61		2 (3)
Sex (male)			54 (79)
Geographical work region in Israel		North	23 (33)
		Jerusalem	12 (18)
		Center	27 (40)
		South	6 (9)
Medical seniority/role		Resident	22 (32)
		Attending Physician	30 (44)
		Unit Director	12 (18)
		Department Director	4 (6)
Primary medical specialty (multiple options were available)		General Surgery	25 (37)
		Trauma	20 (29)
		Neurosurgery/Neurology	4 (6)
		Emergency Medicine	28 (41)
		Pediatrics	14 (21)
Years of experience diagnosing and/or treating individuals following mTBI		Up to 1 year	7 (10)
		1–2 years	6 (9)
		2-5 years	13 (19)
		6-10 years	17 (25)
		10-15 years	11 (16)
		More than 16 years	14 (21)
On average, how many individuals following mTBI do you see per week?		0-2	25 (37)
		3-5	19 (28)
		6-10	16 (23)
		10-15	4 (6)
		More than 16	4 (6)

Values: n (%). mTBI- mild traumatic brain injury

Most respondents were male (79%), between 25 and 40 years old (84%), and senior physicians (68%). The majority have a medical specialty of general surgery and/or trauma surgery (66%). About half of the respondents treat only adults (*n* = 33, 48%), 20% (*n* = 13) treat only children, and 32% (*n* = 22) treat both children and adults.

The majority of respondents (*n* = 38, 56%) received specific training in diagnosing and/or treating patients after mTBI only during medical school. Only 30% (*n* = 20) received specific training in diagnosing and/or treating patients after mTBI in the last five years.

**Fig. 1:**
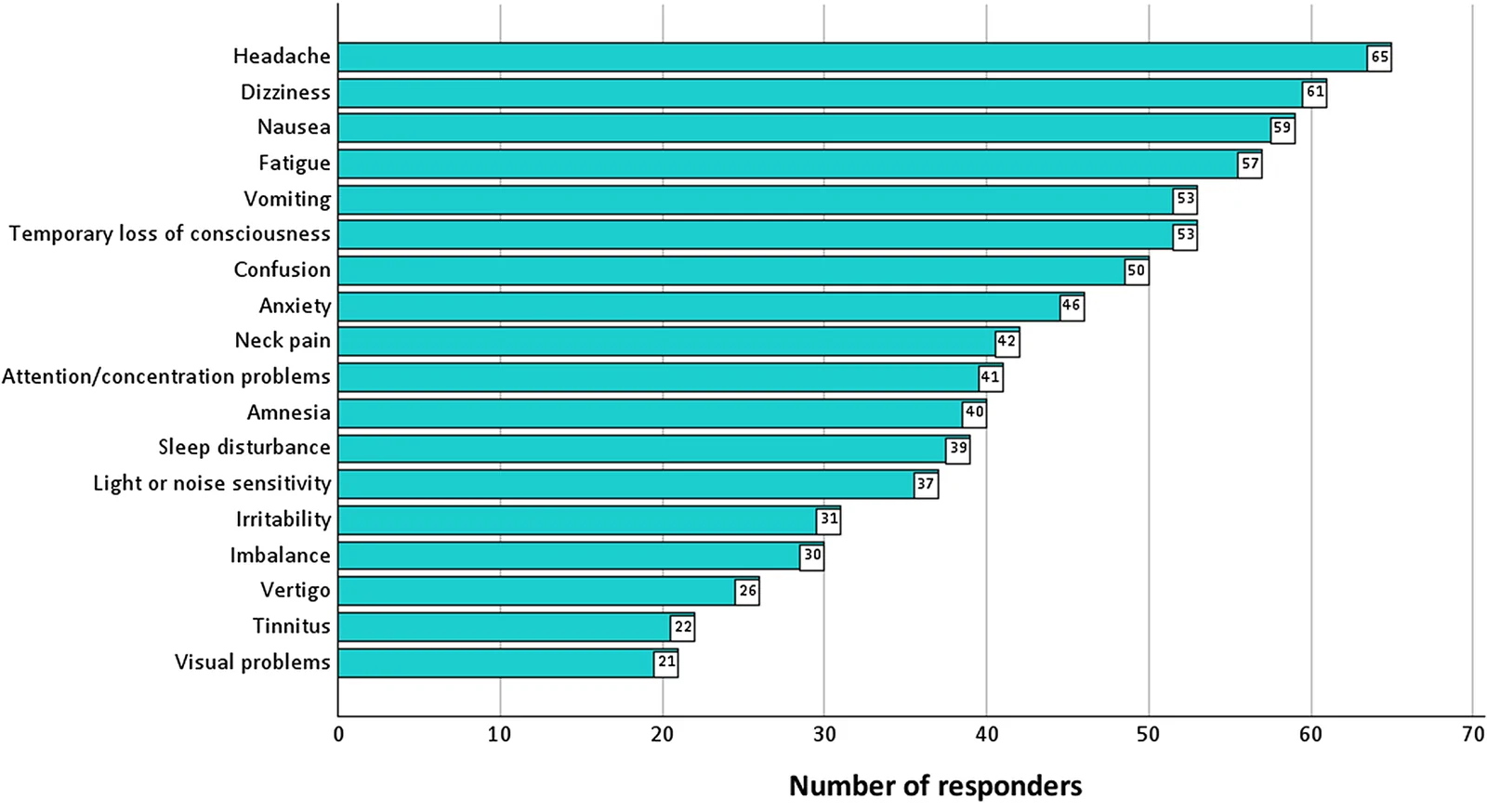
Recognition of symptoms that may occur after mild Traumatic Brain Injury. The numbers at the end of each bar indicate the number of respondents who recognized this symptom

Most respondents (*n* = 52, 77%) are aware that children or adults who have experienced a mTBI may experience cognitive, physical, and emotional/behavioral symptoms; 6% (*n* = 4) respond that they may have experienced only physical symptoms, 9% (*n* = 6) only physical and cognitive symptoms, 4% (*n* = 3) only physical and emotional/behavioral symptoms, and 4% (*n* = 3) only cognitive and emotional/behavioral symptoms. Moreover, although approximately 90% recognized common post-mTBI symptoms, such as headaches, dizziness, nausea, and fatigue, only 40–60% identified neck pain, sleep disturbances, balance problems, vision problems or light/noise sensitivity. A summary of symptoms recognition by the respondents is presented in Fig. 1.

The majority (80%) report that they often (*n* = 25, 37%) or sometimes (*n* = 29, 43%) follow the guidelines for managing individuals after mTBI. But, only 50% reported familiar with the common and standard guidelines [7, 8]: 19 (28%) were familiar with the Ontario Neurotrauma Foundation Living Guidelines, four (6%) with the Department of Veterans Affairs/Department of Defense Guidelines, 14 (21%) with the Centers for Disease Control and Prevention Guidelines, seven (10%) with the Consensus Statement on Concussion in Sport Guidelines, and three (4%) with the American Academy of Neurology Guidelines. Twenty-six (38%) reported following their local hospital’s policy guidelines, and 12 (18%) were not familiar with any guidelines.

**Fig. 2:**
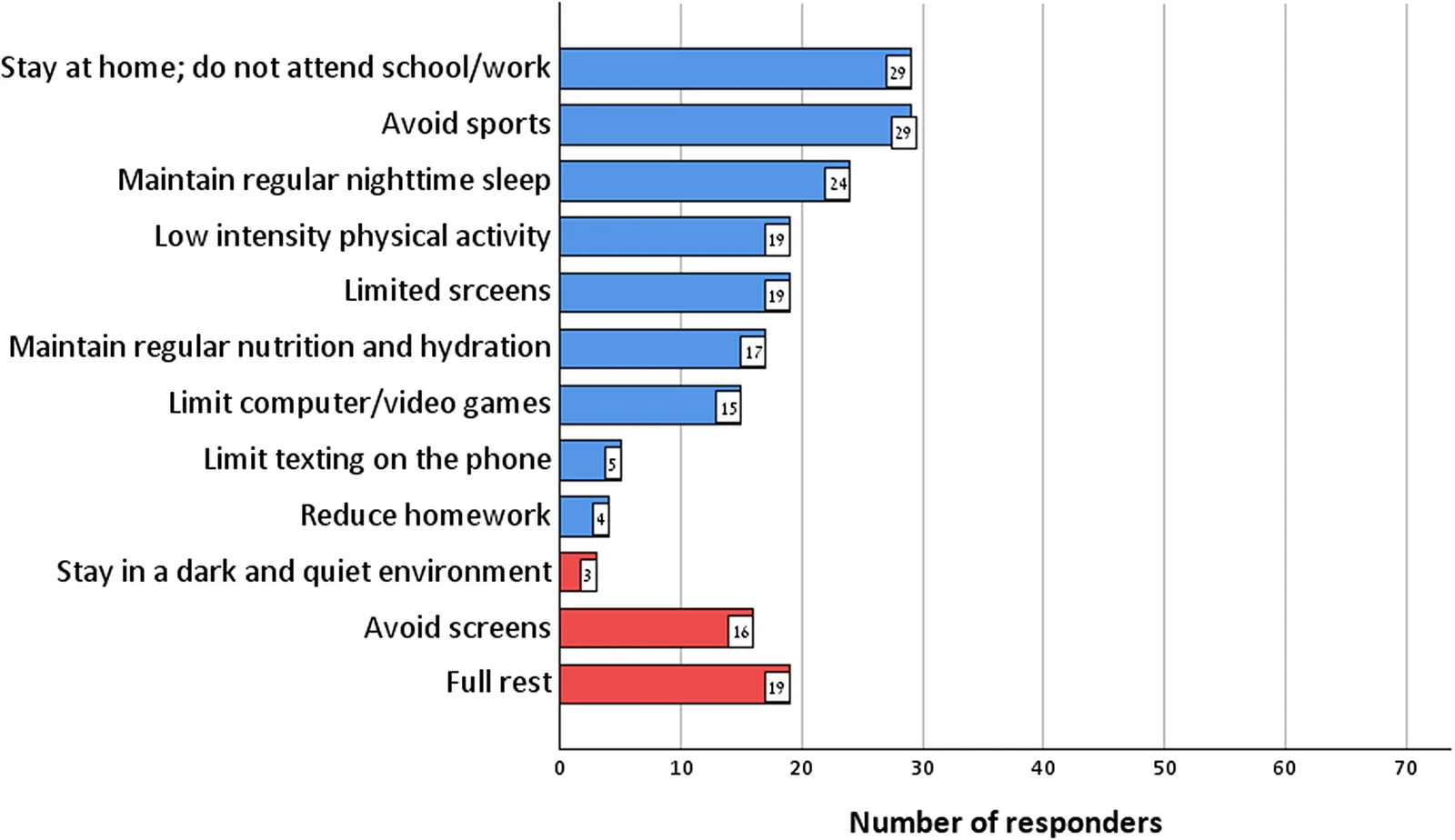
Specific recommendations prescribed after mild Traumatic Brain Injury. The numbers at the end of each bar indicate the number of respondents.Blue color- recommended instructions following mild traumatic brain injury according to the up-to-date guidelines. Red color- instructions no longer recommended following mild traumatic brain injury according to the up-to-date guidelines [6]

Half of the respondents (50%, *n* = 34) prescribe recommendations that include both physical and cognitive rest. A summary of specific recommendations prescribed by the respondents is presented in Fig. 2.

Most respondents recommended activity restriction after mTBI (43% advised staying home/avoiding school/work/sports), whereas fewer provided guidance on screen-time limits (28%), sleep hygiene (35%), nutrition/hydration (25%), or early low-intensity physical activity (28%). Notably, 28% still advised complete rest and 4% recommended a dark/quiet environment, while 7% (*n* = 5) reported providing no recommendations. A quarter of the respondents (24%) provided these recommendations for the first two days after the injury, 40% for 3–5 days after the injury, 18% for more than a week, and 18% with no specific time or depending on the participant’s complaints. Only 21% (*n* = 14) provide patients following mTBI with condition-specific information/education sheets before discharge.

Table 2 summarizes discharge instructions regarding medical follow-up and return-to-sport/school/work protocols.

**Table 2:** Question about discharge instructions for return-to-sport/school/work protocols and medical follow-up

How often do you provide patients with return-to-sport/play protocols guidance before ED/ward discharge?	Never	16 (23)
	Rarely	17 (25)
	Sometimes	12 (18)
	Often	12 (18)
	Always	11 (16)
How often do you provide patients with return-to-school/work protocols and accommodations before ED/ward discharge?	Never	13 (19)
	Rarely	14 (21)
	Sometimes	15 (22)
	Often	18 (26)
	Always	8 (12)
Do you recommend medical follow-up for children (ages 0–18) after concussion/mTBI?	Never	9 (13)
	Rarely	12 (18)
	Sometimes	6 (9)
	Often	8 (12)
	Always	33 (48)
Do you recommend medical follow-up for adults (ages 18+) after concussion/mTBI?	Never	3 (4)
	Rarely	14 (21)
	Sometimes	12 (18)
	Often	6 (9)
	Always	33 (48)
How often do you directly refer patients after concussion/mTBI to a specialist in concussion/mTBI care?	Never	27 (40)
	Rarely	20 (29)
	Sometimes	9 (13)
	Often	4 (6)
	Always	7 (10)
	Did not respond	1 (2)

Values: n (%). mTBI- mild traumatic brain injury

Although 42 (62%) reported recommending a gradual, stepwise return to activity, only 38% provided instructions on return-to-activity/sport protocols, and 32% on return-to-school/work protocols. Furthermore, only 66% (*n* = 45) always or often recommend medical follow-up for children or adults; 60% (*n* = 41) for children, and 57% (*n* = 39) for adults. In addition, only 16% always-often refer their patients after mTBI to a specialist in concussion/mTBI care. The main reason (47%) was a lack of awareness that such a specialty existed. When referring, most refer to a family physician (55%), a pediatrician (30%), a neurologist (38%), or a neurosurgeon (20%). Very few (up to 10%) refer to health professionals such as neuropsychologists, physiotherapists, or occupational therapists. Only two respondents (3%) refer patients to a specialized mTBI clinic.

**Fig. 3:**
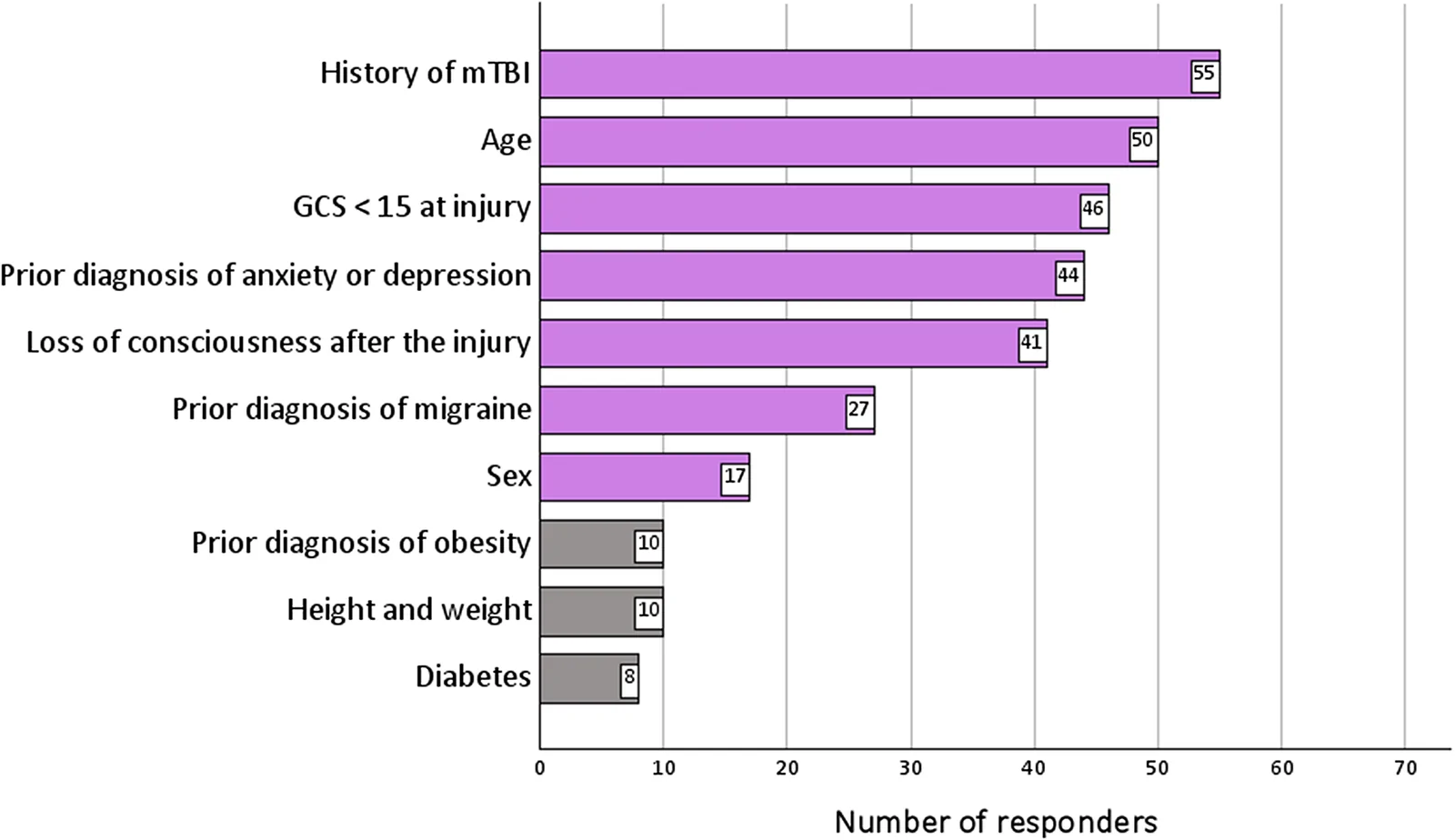
Recognition of risk factors for persistent post-concussion symptoms after mTBI. mTBI- mild traumatic brain injury, GCS- Glasgow Coma Scale.The numbers at the end of each bar indicate the number of respondents.Purple color- Identifying or likely risk factors for persistent post-concussion symptoms. Gray color- factors are not considered risk factors for persistent post-concussion symptoms

Regarding the definition of prolonged recovery and PPCS, 22% (*n* = 15) knew that the timeframe for normal recovery is one month or longer for all. A summary of recognition risk factors for prolonged recovery and PPCS is presented in Fig. 3.

Most respondents recognize previous mTBI (81%), age (73%), Glasgow Coma Scale < 15 (68%), prior diagnosis of anxiety or depression (64%), and loss of consciousness after the injury (60%) as risk factors for prolonged recovery and PPCS. But only 25% recognize sex, and 40% prior diagnosis of migraine. Moreover, some recognized obesity, height and weight and diabetes as risk factors. About half of the respondents (*n* = 30, 44%) always or often instruct patients to seek further care if they develop prolonged recovery and PPCS. It is important to note that 22% of respondents did not follow their mTBI patients for more than two weeks; therefore, they did not answer that question. The main reason (38%) for not instructing patients to seek further care if they develop prolonged recovery and PPCS was a lack of awareness that there is a specialist who treats patients with prolonged recovery and PPCS after mTBI. When referring, most refer to a neurologist (65%), or a neurosurgeon (22%). Very few (up to 15%) refer to a physiatrist (rehabilitation physician) or other health professionals, such as a neuropsychologist, physiotherapist, or occupational therapist. Only two respondents (3%) refer patients to a specialized mTBI clinic in cases of prolonged recovery and PPCS.

Regarding analysis of results between physicians who treat children and those who treat adults, only one significant difference was found; physicians treating children significantly more often recommend follow-up care for children than those treating adults (27/35, 77% versus 14/33, 42% respectively; *p* = 0.006). No other significant differences were observed in responses between physicians treating children and those treating adults. In addition, there were no significant differences in responses between residents and senior physicians, nor between physicians practicing in central Israel (center and Jerusalem) and those practicing in peripheral regions (north and south).

## Discussion

This exploratory study aimed to describe knowledge and self-report implementation of up-to-date clinical guidelines recommendations and referral to follow-up care for patients following mTBI among physicians providing emergency and trauma care in Israel. The survey results indicated substantial gaps between current guidelines recommendations and the self-reported practices of physicians providing first-line care in Israel.

Although almost all respondents treat individuals with mTBI on a weekly basis, only half reported being familiar with any of the major international guidelines, and only 30% had received specific mTBI-related training in the last five years. In addition, although the most common symptoms following mTBI, like headache and dizziness, were commonly reported by the respondents, other frequent symptoms such as neck pain, sleep disturbances, balance problems, and light/noise sensitivity, were much less recognized. These patterns align with previous studies from Canada, the US, and Europe [10–14], indicating that despite high-quality clinical practice guidelines for mTBI, their implementation in emergency and acute care settings remains incomplete and inconsistent.

The findings regarding rest recommendations and early management also highlight a misalignment with contemporary recommendations. Recent consensus statements and guidelines now converge on a brief period (24–48 h) of relative rest followed by a structured, symptom-limited, gradual return to cognitive and physical activity [6, 7, 9]. Although about half of the respondents prescribed both physical and cognitive rest, a substantial proportion still endorsed complete rest, screen avoidance, and “dark and quiet room” strategies for several days or more. Prolonged rest is not beneficial and may even worsen outcomes, whereas early activity and guided re-engagement in daily activities are associated with faster recovery and reduced risk of PPCS [6–8]. In addition, only a third of respondents reported using explicit, stepwise return-to-school/work or return-to-sport protocols, which are the standard of care following mTBI across populations (sports, army, civilians, children or adults) [6, 7, 9].

International guidelines consistently recommend that patients receive standardized written discharge instructions, including education about expected symptoms, red-flag warning signs, favorable expectations for recovery, advice on managing specific symptoms, and graded return-to-school/work/sport plans, as a core component of mTBI care [6–9]. However, in the current study, only about 20% of the respondents routinely provide condition-specific written educational materials at discharge. A previous study showed that a simple intervention, including low-cost guideline-based education and discharge instruction templates, can significantly improve discharge readiness after pediatric concussion [24]. Implementing a similar method in the Israeli emergency departments and/or trauma-related acute-care settings may therefore represent a feasible and relevant strategy for the future.

The data on follow-up and referral pathways presented similar trends. Although not every patient with mTBI requires referral for further treatment, approximately 20–30% may develop persistent symptoms. Guidelines therefore recommend medical follow-up to monitor recovery, and referral to interdisciplinary care when clinically indicated [2, 5–9]. In the present study, only approximately 60% of respondents recommended medical follow-up at discharge, and most were unaware of dedicated interdisciplinary mTBI care. These findings indicate gaps in both routine recovery monitoring and referral pathways for patients with prolonged recovery.

Knowledge of PPCS definitions and risk factors was also inconsistent. The recognition of several major risk factors (previous mTBI, age, pre-injury mental health, loss of consciousness after the injury) was in line with international evidence [7–9]. However, under-recognition of female sex and pre-injury migraine as risk factors, and the reporting of unsubstantiated factors such as obesity or diabetes, point to limited knowledge. In addition, about 50% of respondents usually recommend seeking further care if the patient experiences prolonged recovery and PPCS, but most refer to a neurologist or neurosurgeon. Very few refer to a specific rehabilitation medical team or a specialized mTBI clinic as needed in these cases. Given the limited availability of dedicated interdisciplinary mTBI clinics in Israel, recommendations should prioritize clearer referral criteria and coordination across existing community and rehabilitation services, with access to regional interdisciplinary mTBI care for complex or persistent cases.

When exploring responses by patient population, professional seniority, and geographic location, differences were minimal. These findings suggest that the identified gaps in knowledge, guideline familiarity, and management practices are shared across physician subgroups and likely reflect system-level rather than individual-level or specialty-specific issues.

### Policy implications

#### Need for a coordinated national policy for mTBI care

Although exploratory and based on a relatively small sample, the present findings suggest gaps in acute mTBI care in Israel that may require consideration of a more coordinated national approach. Such an approach could help establish a standard of care across the pathway, from acute diagnosis and discharge to follow-up and, when needed, interdisciplinary management. It could include standardized terminology and documentation, written discharge instructions, education about expected symptoms and red-flag signs, recovery expectations, symptom management, return-to-learn/work/sport protocols, and referral criteria for follow-up and specialized mTBI services [6–9]. Previous studies in other countries have shown that the publication of guidelines alone does not guarantee their implementation, particularly in the absence of a national strategy and practical tools to support uptake in busy emergency and trauma settings [10–14]. This challenge may be even greater in Israel, where mTBI has had less public and professional visibility than in North America, where sport-related concussion has driven awareness, education, and structured return-to-learn/work/sport approaches [6,20, 21]. However, evidence suggests that even modest policy changes may lead to better recovery after mTBI [25].

#### National coordination and responsibility

At the national level, the Israeli Ministry of Health could lead the revision of current policies and procedures, standardize coding and documentation, develop practical implementation tools and a stepped-care pathway, and establish funding mechanisms to support implementation. This process should occur in collaboration with specialists in emergency and trauma medicine, neurology, pediatrics, and rehabilitation medicine, and should also define referral pathways to interdisciplinary care when needed. At the local level, hospitals, emergency departments, trauma units, and community health providers could support implementation through staff training, discharge workflows, and referral pathways linking emergency care with community and rehabilitation services. A shared national-local model may therefore be most appropriate, with national leadership supporting the revision of guidelines and procedures, documentation standards, and implementation frameworks, and local services supporting frontline uptake and continuity of care. In addition, the development of accessible interdisciplinary mTBI services should be a shared responsibility, with the Israeli Ministry of Health providing national planning, standards, and funding mechanisms, and rehabilitation centers establishing and delivering these services.

Within the stepped-care pathway, emergency and trauma teams would diagnose mTBI, assess for red flags, and provide written discharge and initial follow-up guidance; patients and caregivers would monitor symptoms and follow graded return-to-activity, school, or work recommendations; family physicians or pediatricians would assess recovery, identify patients at risk for persistent symptoms or experiencing prolonged recovery, and coordinate referral when needed; and rehabilitation teams would provide symptom-targeted interdisciplinary care for patients with persistent or complex symptoms. National quality indicators could assess implementation across this pathway, including discharge guidance and information transfer, primary-care follow-up, and timely referral to interdisciplinary rehabilitation services when indicated.

#### Implementation of guidelines in clinical practice

Even when guidelines are available, implementation in routine clinical practice remains challenging, particularly in busy emergency and trauma settings. A pragmatic initial step may therefore be to prioritize scalable, lower-cost strategies such as standardized discharge materials [24], brief e-learning modules [26], integration of mTBI content into residency curricula [27], and structured referral workflows. At discharge, a structured, evidence-based document should be provided to the patient or caregiver and incorporated into the discharge summary transferred to the family physician or pediatrician. It should include the diagnosis, red flags, expected recovery, symptom-management and graded-return-to-activity recommendations, and the criteria for prolonged recovery and interdisciplinary team follow-up [6–9]. In addition, decision-support tools embedded within electronic medical record systems may help improve adherence to clinical guidelines and facilitate their implementation when integrated into routine workflow [28–30]. In the context of mTBI care, such tools could include discharge templates, smart phrases, referral prompts, and automated reminders for follow-up recommendations. Electronic systems could send an automated SMS or app-based reminder within approximately 48 h of discharge, helping patients or caregivers to review return-to-activity protocols and seek follow-up when indicated. Such reminders could be prioritized for patients at high risk of prolonged recovery. AI-enabled decision-support tools may also represent a future implementation option, for example, by helping identify patients at higher risk for prolonged recovery, prompting clinicians regarding follow-up needs, or supporting more consistent documentation and triage.

#### Economic considerations and policy feasibility

From a policy perspective, one potential rationale for standardized mTBI care is that it may reduce the number of patients who experience prolonged recovery or PPCS, conditions that often require broader, more resource-intensive services [31]. Direct economic evidence regarding implementation of mTBI guidelines is limited. But individuals with PPCS have been associated with greater healthcare utilization and higher long-term costs [32], whereas interdisciplinary mTBI care models have been described as accessible, comprehensive, and potentially cost-saving [31, 32]. For this reason, implementation may reasonably begin with scalable and relatively low-cost interventions, such as standardized discharge materials, targeted training, improved documentation, and referral support tools. Together, these measures may improve the consistency of early mTBI care while reducing downstream burden on the healthcare system.

### Limitations

This study has several limitations. First, the exploratory design and the relatively small sample size, which consists mainly of male trauma/surgical physicians, limit generalizability to other specialties or to community-based clinicians. In addition, dissemination of the survey relied on professional networks and onward sharing, and the total number of clinicians who viewed the invitation is unknown. Second, as the survey was distributed via social media and participation was voluntary, selection bias is possible, as physicians with a greater interest in mTBI might have been more likely to respond. All data are self-reported and reflect perceived rather than observed practice, actual behavior may differ from what the respondents report. Future larger studies are needed at both conceptual and implementation levels, including qualitative studies, to clarify attitudes, perceived barriers, and needs regarding the use of current mTBI guidelines in Israel.

## Conclusion

This is, to our knowledge, the first study to describe knowledge and practice patterns related to mTBI guidelines among physicians providing emergency and trauma care in Israel. The findings indicated substantial gaps between current international guideline recommendations regarding discharge instructions and referral to ongoing care after mTBI, and the self-reported knowledge and practices of emergency and trauma physicians in Israel. Addressing these gaps through coordinated national educational initiatives, system-level implementation strategies, revisions to national protocols, and targeted professional training could enhance the consistency and quality of mTBI care. These exploratory findings also support consideration of clearer referral pathways and the development of accessible interdisciplinary mTBI services for patients with persistent or complex symptoms in Israel.

## Supplementary Information

Below is the link to the electronic supplementary material.

Supplementary Material 1

## Data Availability

The datasets used and/or analysed during the current study are available from the corresponding author on reasonable request.
