# Supplementary material for: Knowledge and practice of evidence-based mild traumatic brain injury guidelines among physicians providing emergency and trauma care in Israel
Source: Isr J Health Policy Res. 2026 Sep 15;15:41. doi: 10.1186/s13584-026-00784-x (PMC13576113; doi:10.1186/s13584-026-00784-x)
Supplement: Supplementary file 1 [file 13584_2026_784_MOESM1_ESM.pdf]

Concussion/Mild Traumatic Brain Injury (mTBI) Referral and Follow-Up Questionnaire

Introduction

This questionnaire examines the knowledge and implementation of up-to-date clinical guidelines for discharge, referral, and follow-up of patients after mild traumatic brain injury (mTBI, also known as concussion) by emergency department physicians in Israel.
The questionnaire consists of two parts: Part A includes general information about the respondent; Part B assesses the respondent's knowledge and implementation of referral and follow-up for concussion/mTBI.
You are under no obligation to complete the questionnaire.

Part A: General Information

1. Age

☐ 25–30 years
☐ 30–40 years
☐ 40–50 years
☐ 50–60 years
☐ 60+

2. Sex

☐ Male
☐ Female
☐ Other

3. Geographical work region in Israel:

☐ Central District
☐ Tel Aviv District
☐ Haifa District
☐ Northern District
☐ Southern District
☐ Jerusalem District

4. Medical seniority/role:

☐ Resident
☐ Attending/Staff Physician

☐ Unit Director

☐ Department Director

5. Primary medical specialty:

☐ Trauma

☐ General Surgery

☐ Neurosurgery

☐ Neurology

☐ Orthopedics

☐ Emergency Medicine

☐ Internal Medicine

☐ Pediatric Surgery

☐ Pediatrics

☐ Other, specify: ___________

6. Years of experience diagnosing and/or treating children and/or adults with concussion/mTBI:

☐ Up to 1 year

☐ 1–2 years

☐ 2–5 years

☐ 5–10 years

☐ 10–15 years

☐ More than 16 years

7. On average, how many children and/or adults with concussion/mTBI do you see per week?

☐ 0–2

☐ 3–5

☐ 6–10

☐ 11–15

☐ 16–20

☐ 21–25

☐ 25–30

☐ More than 31

8. Do you diagnose and/or treat children and/or adults with concussion/mTBI in your work?

☐ Adults
☐ Children
☐ Adults and children
☐ No (if “No”, do not proceed with the questionnaire)

Part B: Referral and Follow-Up Concussion/mTBI patients

1. Children or adults after a concussion/mTBI may experience various symptom types. Select the symptom types they may have:

☐ Cognitive symptoms
☐ Physical symptoms
☐ Emotional/behavioral symptoms
☐ Physical and cognitive symptoms
☐ Physical and emotional/behavioral symptoms
☐ Cognitive and emotional/behavioral symptoms
☐ Cognitive, physical, and emotional/behavioral symptoms

2. Mark all symptoms from the list that may occur after concussion/mTBI (select all that apply):

☐ Headache
☐ Dizziness
☐ Confusion
☐ Nausea
☐ Vomiting
☐ Vertigo
☐ Amnesia
☐ Tinnitus
☐ Neck pain
☐ Sensitivity to light or noise
☐ Temporary loss of consciousness
☐ Anxiety
☐ Irritability
☐ Attention or concentration problems
☐ Sleep disturbance
☐ Imbalance
☐ Visual problems
☐ Fatigue

3. In the past 10 years, several guidelines have been published for managing children or adults after concussion/mTBI. Do you follow these guidelines?

☐ No
☐ Rarely

☐ Sometimes
☐ Often
☐ Always

4. From the list below, which guidelines are you familiar with? (select all that apply)

☐ Ontario Neurotrauma Foundation Guideline for Concussion/Mild Traumatic Brain Injury & Persistent Symptoms (Living guidelines)
☐ Department of Veterans Affairs/Department of Defense Clinical Practice Guideline for the Management of Concussion-Mild Traumatic Brain Injury (2021)
☐ Centers for Disease Control and Prevention Guideline on the Diagnosis and Management of Mild Traumatic Brain Injury Among Children (2023)
☐ Consensus Statement on Concussion in Sport, the 5th International Conference on Concussion in Sport (2023)
☐ Guideline Development Subcommittee of the American Academy of Neurology (2014)
☐ Hospital/practice policy
☐ Other: _______________
☐ None

5. Do you prescribe rest recommendations to patients after concussion/mTBI?

☐ Yes, physical rest
☐ Yes, cognitive rest
☐ Yes, both physical and cognitive rest
☐ Yes, both physical and cognitive rest, depend on the patient's symptoms
☐ Depends on the patient
☐ Depends on the patient's symptoms
☐ Depends on the patient's age
☐ I do not prescribe rest recommendations

6. Which additional recommendations do you provide for the days after ED/ward discharge? (select all that apply)

☐ Stay at home; do not attend school/work
☐ Avoid screens
☐ Avoid sports
☐ Low intensity physical activity
☐ Full rest
☐ Limit texting on the phone
☐ Limit screen time
☐ Reduce homework
☐ Stay in a dark and quiet environment
☐ Maintain regular nighttime sleep

☐ Maintain regular nutrition and hydration
☐ All of the above
☐ None of the above
☐ Don't know
☐ Other: ______________

7. For how long do you prescribe initial rest and/or the additional recommendations?

☐ First 24 hours after injury
☐ 24–48 hours after injury
☐ 3–5 days after injury
☐ 1 week after injury
☐ 2 weeks after injury
☐ No exact timeframe; depends on the patient's condition
☐ Don't know

8. What return recommendations do you provide for school/sport/work, etc., after concussion/mTBI?

☐ Return after clinical evaluation
☐ Return after symptoms resolve
☐ Gradual, stepwise return based on symptoms
☐ Gradual return
☐ Referral to a physician to authorize return
☐ Return according to the demands of the specific activity

9. How often do you provide patients with return-to-sport/return-to-play protocol guidance before ED/ward discharge?

☐ Never
☐ Rarely
☐ Sometimes
☐ Often
☐ Always

10. How often do you provide patients with return-to-school/work protocols and accommodations before ED/ward discharge?

☐ Never
☐ Rarely
☐ Sometimes
☐ Often
☐ Always

11. Do you recommend medical follow-up for children (ages 0–18) after concussion/mTBI?

☐ Never

☐ Rarely

☐ Sometimes

☐ Often

☐ Always

12. Do you recommend medical follow-up for adults (ages 18+) after concussion/mTBI?

☐ Never

☐ Rarely

☐ Sometimes

☐ Often

☐ Always

13. How often do you directly refer patients after concussion/mTBI to a specialist in concussion/mTBI care?

☐ Never

☐ Rarely

☐ Sometimes

☐ Often

☐ Always

14. If you refer: To which of the following specialists do you refer? (select all that apply)

☐ Family physician

☐ Pediatrician

☐ Neurologist

☐ Physiatrist (Rehabilitation physician)

☐ Neuropsychologist

☐ Sports physician

☐ Neurosurgeon

☐ Psychiatrist

☐ Physiotherapist

☐ Occupational therapist

☐ Other: ___________

15. If you do not refer, why do you choose not to refer to a concussion/mTBI specialist?

☐ No specialists in my geographical area

☐ Prefer to follow my patients myself

☐ No need for a concussion/mTBI specialist
☐ No specific reason
☐ I was unaware that such a specialty existed.
☐ Not applicable, as I always refer patients with a concussion/mTBI to a specialist.

16. Do patients with concussion/mTBI in your department receive condition-specific information/education sheets before discharge?

☐ Yes
☐ No
☐ Don't know

17. Patients experiencing moderate–severe symptoms or those unable to resume regular activities are defined as having Persistent Post-concussion symptoms (PPCS). In your opinion, what is the timeframe for defining PPCS?

☐ After 2 weeks for adults and 1 month for children/adolescents
☐ After 2 weeks for the general population
☐ After 1 month for the general population
☐ After 2 weeks for children/adolescents and 1 month for adults

18. From the list below, select all risk factors for developing PPCS:

☐ Younger age
☐ Older age
☐ History of concussion/mTBI
☐ Sex
☐ Prior diagnosis of obesity
☐ Prior diagnosis of diabetes
☐ Glasgow Coma Scale (GCS) < 15 at injury
☐ Prior diagnosis of migraine
☐ Loss of consciousness after the injury
☐ Prior diagnosis of anxiety or depression

19. Do you instruct your patients to seek further care if they develop PPCS?

☐ Never
☐ Rarely
☐ Sometimes
☐ Often
☐ Always
☐ Usually do not follow patients beyond two weeks

20. If you refer (for PPCS treatment): To which specialists do you refer?

☐ Neurologist
☐ Physiatrist (Rehabilitation physician)
☐ Neuropsychologist
☐ Sports physician
☐ Neurosurgeon
☐ Psychiatrist
☐ Physiotherapist
☐ Occupational therapist
☐ Other: __________

21. If you do not refer (for PPCS treatment): Why do you choose not to refer to a PPCS specialist?

☐ No specialists in my geographical area
☐ Prefer to follow my patients myself
☐ No need for a concussion/mTBI specialist
☐ No specific reason
☐ I was unaware that such a specialty existed.
☐ Not applicable, as I always refer patients with a concussion/mTBI to a specialist.

22. Have you received specific training in diagnosing and/or treating patients after concussion/mTBI?

☐ Within the past year
☐ Within the past 3 years
☐ Within the past 5 years
☐ Within the past 10 years
☐ More than 10 years ago
☐ Only training during medical school

Thanks for filling out this questionnaire!
